# Examining the impact of adolescent social isolation on oxycodone sensitization

**DOI:** 10.1007/s00213-025-06914-8

**Published:** 2025-10-09

**Authors:** Erin A. English, Lisa A. Briand

**Affiliations:** 1 Department of Psychology and Neuroscience, Temple University, Weiss Hall, 1701 North 13th Street, Philadelphia, PA 19122, USA; 2 Neuroscience Program, Temple University, Philadelphia, PA 19122, USA

**Keywords:** Adolescence, Social isolation, Oxycodone, Sensitization

## Abstract

**Rationale:**

Chronic stress and social isolation during adolescence can disrupt normal development and increase substance use behaviors in adulthood.

**Objectives:**

The impact of adolescent social isolation stress on stimulant behaviors has been identified in a mouse model. The impact of adolescent social isolation on opioid behaviors is less clear. We assess the effect of adolescent social isolation on oxycodone sensitization across sexes and light phase.

**Methods:**

Mice were either group-housed or socially isolated at weaning and remained in these housing conditions until adulthood. Male and female mice in both housing conditions received 5 days of daily injections of oxycodone (5 mg/kg) during either the light or dark phase. A subset of mice remained in their home cage for five additional days prior to a final oxycodone challenge.

**Results:**

When tested during the light phase male mice in both housing conditions exhibit oxycodone sensitization to a 5 mg/kg dose, while female mice do not. In contrast, when tested during the dark phase, none of the groups exhibit oxycodone sensitization. In fact, female mice develop tolerance to the locomotor activating effects of oxycodone after five daily injections. Following five days in the home cage, group housed females continued to exhibit tolerance to the locomotor effects of oxycodone, while socially isolated females do not. Additionally, socially isolated females exhibit a larger conditioned response to the arena during the habituation phase than animals in any of the other groups.

**Conclusions:**

Sensitization is a light phase dependent process in male mice. Female mice do not sensitize to 5 mg/kg oxycodone. Adolescent social isolation stress influences the development of tolerance, not sensitization. Oxycodone induces sex-specific effects on locomotion during habituation phase.

## Introduction

Adolescent development and experiences promote the foundation for behavior in adulthood. Stressors during adolescence, including those social in nature, can promote maladaptive behaviors in adulthood. Adverse childhood experiences, including social isolation, are linked to increased impulsivity and altered prefrontal cortex activity, which are proposed to promote and maintain drug taking behaviors in adolescence and adulthood (de Costa Macedo et al. 2025). Thus, chronic stress, especially during early life, has long been proposed as a risk factor for developing psychiatric disorders including substance use disorder (SUD) both in adolescence and adulthood. Furthermore, opioid use disorder (OUD) is highly correlated with a history of childhood trauma, especially in female populations, as well as psychological trauma in adulthood (Jeffries-Baxter et al. 2024; de Costa Macedo et al. 2025). Adverse childhood experiences are also broadly associated with a later need for analgesic opioid use and altered analgesic responses leading to higher misuse and abuse liability (Senaratne et al. 2025). Differentially methylated opioid receptor genes could provide a mechanism for how childhood stress impacts opioid responses (Gescher et al. 2024). However, these broad human studies present a preclinical need to further parse out how the timepoints at which social stressors are introduced and impact opioid behaviors. The need for preclinical studies of stress-prompted development of SUDs is highlighted by the severity in which SUDs manifest. SUD and substance misuse can be fatal in some instances of overdose. Drug overdose, including opioid overdose, is increasing in the United States and has been classified as the leading cause of accidental death with deaths from opioid overdose representing one of the sharpest inclines in recent decades (Garnett and Minino 2024; Burns et al. 2025).

Adolescent stress and adverse childhood experiences altering alcohol and stimulant behaviors have been well documented in clinical and preclinical studies. Following early life and adolescent stress, rodents, especially female rodents, increase ethanol consumption (McElroy et al. 2023; Parks et al. 2024). Previously, our lab developed a mouse model of adolescent social isolation that increases motivation for cocaine and cocaine seeking in both males and females (Fosnocht et al. 2019). There is also some evidence for stress-primed vulnerability to rodent opioid addiction has been studied specifically using heroin (Singh et al. 2022). Interestingly, models of early life adversity and social isolation have developed bidirectional sex effects in affective and addiction-related behaviors relating to morphine self-administration (Ordoñes Sanchez et al. 2021; Wang et al. 2022). Adolescent social isolation in rodents potentially alters drug responses through further disruption of the dopaminergic and endogenous opioid systems (Karkhanis et al. 2014, 2015, 2016, 2019; Yorgason et al. 2016; Zhang et al. 2024). However, more research is needed to understand the way this model interacts with different opioids, specifically oxycodone, and the molecular underpinnings to this interaction.

Important, but often overlooked, biological variables contributing to drug phenotypes relates to circadian rhythms and time of day. From a methodological standpoint, the time of day testing is initiated may influence responses to both natural and drug reward (Hyytiä and Sinclair 1990; Coffey et al. 2018; Acosta et al. 2020; DePoy et al. 2021). Specifically, rodents more readily self-administer opioids during the dark phase which is the animals’ active phase (Coffey et al. 2018). It is unclear how light phase may alter oxycodone phenotypes; therefore, this study aims to examine the impact of adolescent social isolation stress on oxycodone sensitization and tolerance behaviors across light phases using both male and female mice.

Substance-induced behavioral locomotion sensitization is a phenomenon observed as an increased behavioral response to a substance following repeated exposure (Kilbey and Ellinwood 1977; Robinson 1984). Sensitization is paired conversely with habituation or tolerance which is a phenomenon observed as a decreased behavioral response to repeated substance exposure. The induction of behavioral sensitization has been well documented across drug classes though mainly using stimulants like cocaine or methamphetamine and the opioid morphine (Kilbey and Ellinwood 1977; Robinson 1984; Drew and Glick 1988; Gaytan et al. 1999, 2000; Ericson et al. 2010; Niikura et al. 2013). Given that the behavioral output in a sensitization paradigm changes across time points, we view sensitization as a measure of drug-induced behavioral plasticity and a proxy measure of synaptic plasticity following substance exposure. This behavioral measure of plasticity can then be used to examine the ability for the animals within our adolescent social isolation model to undergo plasticity and could potentially speak to the resilience and vulnerability to oxycodone behaviors.

We aim to examine differences in drug-induced behavioral plasticity and long-term oxycodone responses in mice who have undergone adolescent social isolation. We hypothesize that adolescent social isolation will alter the long-term behavioral response to oxycodone potentially in a sex-specific manner.

## Methods

### Animals

Male and female C57BL/6J mice were bred in house. Mice were randomly assigned to isolation (single-housed) or group-housed (3–5 mice per cage) conditions at weaning (PND 21). Mice were kept in these conditions until testing began at PND 60 which represents the start of adulthood. Mice were housed were with age-matched conspecifics of the same sex. All animal care facilities were temperature and humidity controlled. Mice in the light phase experiments were housed in a room with a 12-hour light/dark cycle: (lights on at 7:00 a.m and off at 7:00 p.m. Mice in the dark phase experiments were housed in a room with a reversed light cycle (lights on at 7:00 p.m. and off at 7:00 a.m.). Animals received food and water *ad libitum*. All procedures were approved by the Temple University Animal Care and Use Committee.

### Drugs

Oxycodone hydrochloride was obtained from the National Institute of Drug Abuse Drug Supply Program (Bethesda, MD) and dissolved in sterile 0.9% saline.

### Oxycodone sensitization

Sensitization experiments were conducted in a white, plastic, open field arena (35 × 35 × 24 cm) with corncob home cage bedding (Anderson Bed-o-Cobs 1/8”) under red light (8.2–12.4 lx). Behavioral testing occurred between ZT1 and ZT7 in the light phase and ZT13 and ZT19 in the dark phase. Individual mice were tested at the same time each day. Experiments were conducted for five consecutive days with a subset of animals receiving an additional test for expression following five days in the home cage without testing or drug administration. On each test day, after 15 min of habituation to the testing room, animals were first placed in the arena and locomotor activity was measured 30 min prior to any drug injection (habituation phase). This habituation phase controls for any conditioned locomotor effects of the arena prior to the administration of oxycodone. Following the habituation phase, mice were injected with oxycodone (5 mg/kg, i.p.) and immediately returned to the arena. Animals remained in the arena for a 60-minute period to assess locomotor activity post-injection. Total distance traveled was tracked and recorded during the habituation and post-injection phases using ANY-Maze Video Tracking System (Stoelting Co., Version 7.4). Locomotor activity data was collected as distance traveled within five-minute bins and summed to the total distance travelled.

### Data analysis

All statistical analysis was conducted using GraphPad Prism 10. Area under the curve analysis was conducted for habituation and post-injection data by day. Area under the curve output group comparisons were then analyzed using two-way ANOVA. Area under the curve between days was analyzed using a repeated measures two-way ANOVA. Locomotor activity in five-minute bins was analyzed using a three-way ANOVA. Sidak’s post-hoc testing was used when appropriate. Statistical significance was set at α = 0.05.

## Results

### Habituation phase locomotor activity in the light phase (Fig. 1a)

**Fig. 1:**
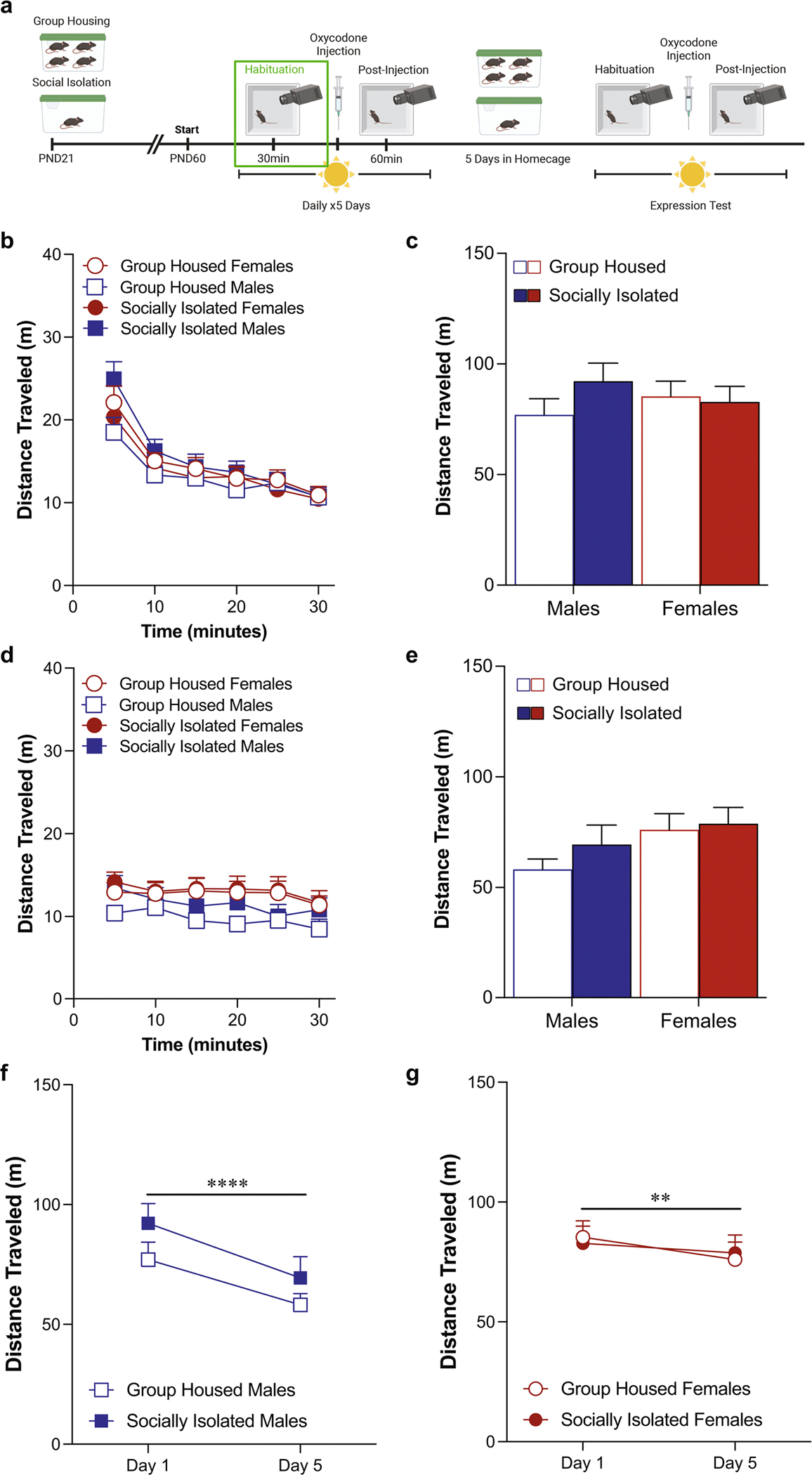
Sex differences in locomotor behavior appear during the habituation phase when mice are tested during the light phase. Schematic of experimental timeline with habituation phase outlined (**A**). Adolescent social isolation does not alter basal locomotor activity (**B**, **C**) during the first habituation testing session. Adolescent social isolation does not alter locomotor activity (**D**, **E**) during the habituation session on the fifth test day. Both male (F, ****p < 0.0001) and female (**G**, **p = 0.02) mice exhibit locomotor habituation on the fifth test day. Locomotor activity is represented through distance traveled within five-minute bins (**B**, **D**) and total distance traveled (**C**, **E**). Data are presented as averages with SEM. (GH females *n* = 13 (**A**-**B***n* = 12), GH males *n* = 13 (A-B *n* = 12), ASI females *n* = 13, ASI males *n* = 13)

On the first day of testing, adolescent social isolation did not alter basal locomotor activity in a novel environment over the course of the habituation phase (Fig. 1b: three-way ANOVA: effect of housing: *F*(1,46) = 0.26, *p* = 0.61, effect of sex: *F*(1,46) = 0.003, *p* = 0.95; effect of time: *F*(2.988,137.4) = 108.2, *p* < 0.0001). However, there was a time by housing condition by sex interaction in locomotor activity [interaction: *F*(2.988,137.4) = 3.64, *p* = 0.015), an effect that is most likely driven by the locomotor activity during the first five minutes of the habituation phase. When we analyze this time block separately it reveals a significant housing condition by sex interaction (Supplemental Fig. 1; two-way ANOVA: effect of housing: *F*(1,46) = 1.43; *p* = 0.24; effect of sex: *F*(1,46) = 0.06; *p* = 0.81 interaction: *F*(1,46) = 4.27; *p* = 0.045; Sidak’s post-hoc male group housed vs. male social isolation: *p* = 0.051). When we calculate the total distanced traveled on Day 1 this effect washes out and there are no differences between the groups (Fig. 1b: two-way ANOVA: effect of housing: *F*(1,46) = 0.26, *p* = 0.61, effect of sex: *F*(1,46) = 0.003, *p* = 0.95, interaction: *F*(1,46) = 1.41, *p* = 0.24). On the fifth day of oxycodone administration, there are no significant effects of either housing condition or sex on locomotor behavior (Fig. 1d: three-way ANOVA: effect of housing: *F*(1,48) = 0.93, *p* = 0.34, effect of sex: *F*(1,48) = 3.57, *p* = 0.07; effect of time: *F*(3.998,191.9) = 6.01, *p* = 0.0001; Fig. 1e: two-way ANOVA: effect of housing: *F*(1,48) = 0.93, *p* = 0.34, effect of sex: *F*(1,48) = 3.57, *p* = 0.07; interaction: *F*(1,48) = 0.34, *p* = 0.56). Comparing the change in locomotor activity on the first day of habituation to the fifth day, male mice exhibited a decrease, regardless of housing condition (Fig. 1f: two-way ANOVA, effect of test day: *F*(1,24) = 25.09, *p* < 0.0001, effect of housing condition: *F*(1,24) = 1.85, *p* = 0.19). Our female mice also show significant habituation on the fifth day of testing (Fig. 1 g: two-way RM ANOVA, effect of test day: *F*(1,24) = 5.55, *p* = 0.02, effect of housing condition: *F*(1,24) = 0.0002, *p* = 0.99). If we run a three way ANOVA on this data, it reveals a significant test day by sex interaction with only the male mice exhibiting an effect of test day [test day x sex interaction: *F*(1,48) = 8.0, *p* = 0.007; Sidak’s post-hoc effect of day in group housed males: *p* = 0.012; effect of day in socially isolated males: *p* = 0.001; effect of day in group housed females: *p* = 0.87; effect of day in socially isolated females: *p* = 0.99).

### Oxycodone-induced locomotor activity in the light phase (Fig. 2a)

**Fig. 2:**
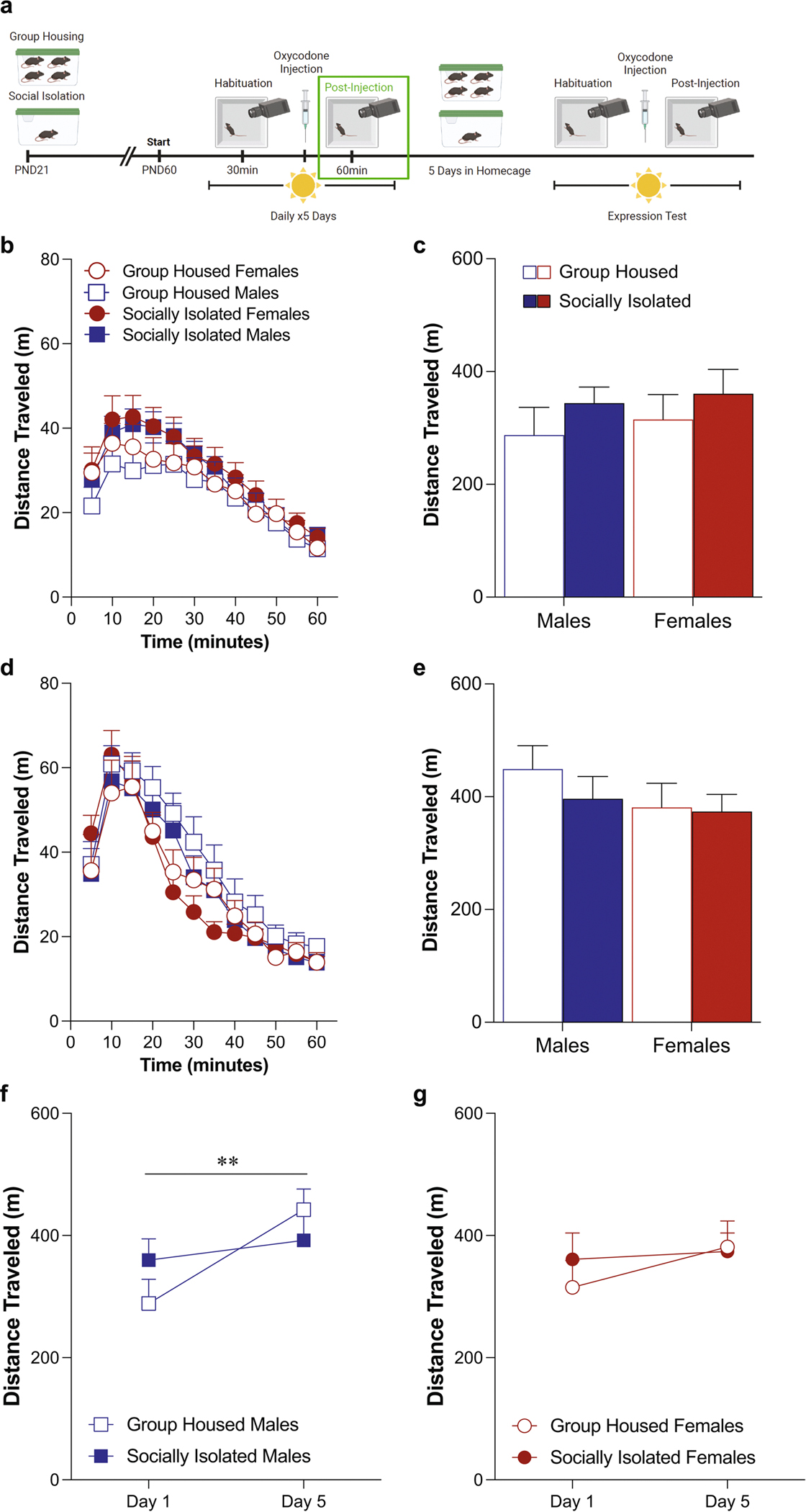
Male mice exhibit locomotor sensitization to oxycodone administered in the light phase, while female mice do not. Schematic of experimental timeline with post-injection phase outlined (**A**). Adolescent social isolation does not alter the acute locomotor response to oxycodone (**B**, **C**) Adolescent social isolation does not alter locomotor activity after the fifth oxycodone administration (**D**, **E**). Male mice exhibit sensitization to oxycodone on the fifth test day (**F**). Female mice do not exhibit sensitization on fifth test day (**G**). Locomotor activity is represented through distance traveled within five-minute bins (**B**, **D**) and total distance traveled (**C**, **E**). Data are presented as averages with SEM. (GH females *n* = 13, GH males *n* = 13, ASI females *n* = 13, ASI males *n* = 13)

Adolescent social isolation did not alter the acute locomotor response to oxycodone (5 mg/kg) on day 1, when mice were tested during their light phase (Fig. 2b, c: three-way ANOVA: effect of housing condition: *F*(1,48) = 1.50, *p* = 0.23; effect of sex: *F*(1,48) = 0.28, *p* = 0.60; effect of time: *F*(3.199,153.5) = 75.24, *p* < 0.0001). While there were still no main effects of housing condition on locomotor activity after the fifth day of oxycodone administration (Fig. 2d, e: three-way ANOVA: effect of housing condition: *F*(1,48) = 0.60, *p* = 0.44; effect of sex: *F*(1,48) = 1.39, *p* = 0.25; effect of time: *F*(11,528) = 83.12, *p* < 0.0001), there was a time by sex interaction [*F*(11,528) = 2.14, *p* = 0.017) but none of the post-hoc comparisons were significantly different. Comparing the change in locomotor response to oxycodone on day 1 vs. day 5, male mice in both housing conditions exhibit sensitization, increasing their locomotor response on day 5 (Fig. 2f: two-way ANOVA: effect of test day: *F*(1,24) = 8.90, *p* = 0.006, effect of housing condition: *F*(1,24) = 0.046, *p* = 0.83). However, sensitization did not occur in female mice (Fig. 2 g: two-way ANOVA: effect of test day: *F*(1,24) = 2.10, *p* = 0.16, effect of housing condition: *F*(1,24) = 0.15, *p* = 0.71).

### Habituation phase locomotor activity during the dark phase (Fig. 3a)

**Fig. 3:**
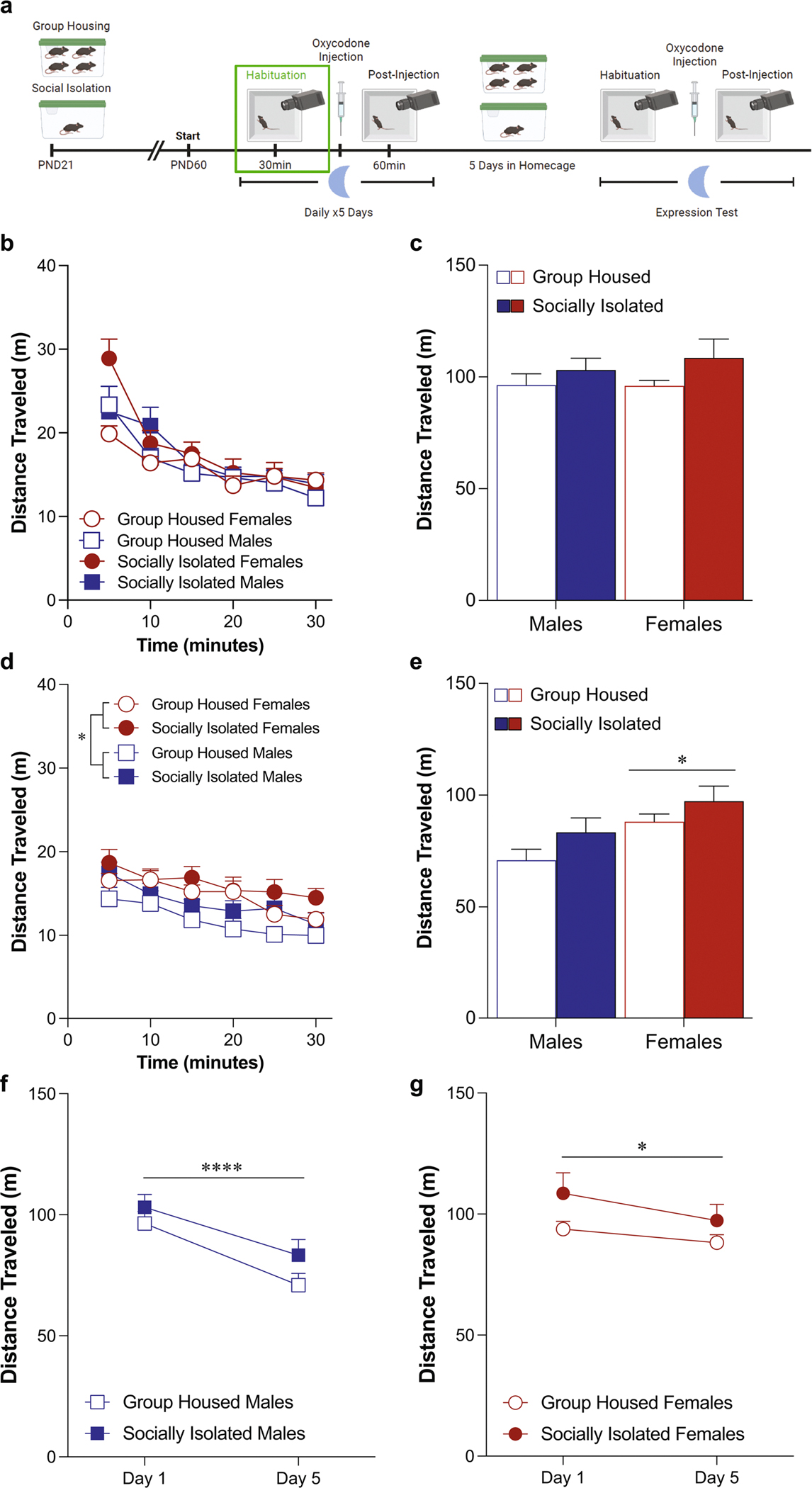
Sex differences in locomotor behavior appear during the habituation phase when mice are tested during the dark phase. Schematic of experimental timeline with habituation phase outlined (**A**). Adolescent social isolation does not alter basal locomotor activity (**B**) or total distance traveled (**C**) during the first habituation testing session. Female mice exhibit higher levels of locomotion compared to male mice across the fifth testing session (**D**, **E**, *p = 0.01). Both male (**F**, ****p < 0.0001) and female (**G**, *p = 0.02) exhibit locomotor habituation on the fifth testing day. Locomotor activity is represented through distance traveled within five-minute bins (**B**, **D**) and total distance traveled (**C**, **E**). Data are presented as averages with SEM. (GH females *n* = 7, GH males *n* = 9, ASI females *n* = 9, ASI males *n* = 9)

When examining habituation behavior in mice tested during their dark phase, while we did not detect an overall effect of housing condition on locomotor behavior on day 1 (Fig. 3b: three-way ANOVA: effect of housing condition: *F*(1,30) = 2.55, *p* = 0.12, effect of sex: *F*(1,30) = 0.18, *p* = 0.67; effect of time: *F*(3.906,117.2) = 55.4, *p* < 0.0001; Fig. 3c: two-way ANOVA: effect of housing condition: *F*(1,30) = 2.55, *p* = 0.12, effect of sex: *F*(1,30) = 0.18, *p* = 0.67), we did detect a time by housing condition by sex interaction [*F*(3.906,117.2) = 5.05, *p* = 0.0009). As with the habituation data from the light phase, this effect appears to be driven by the first five minutes. However, in contrast to the light phase data which was driven by an increase in locomotor activity in the socially isolated males, this effect is driven by an increase in the socially isolated females (Supplemental Fig. 2: two-way ANOVA: effect of housing condition: *F*(1,30) = 5.13, *p* = 0.03; effect of sex: *F*(1,30) = 0.65, *p* = 0.43; interaction: *F*(1,30) = 7.33, *p* = 0.01; Sidak’s post-hoc group housed females vs. socially isolated females: *p* = 0.004). On the fifth day, while there was no effect of housing condition on locomotor behavior, female mice exhibited higher levels of locomotion compared to male mice across the session (Fig. 3d: three-way ANOVA: effect of housing condition: *F*(1,30) = 3.48, *p* = 0.07; effect of sex: *F*(1,30) = 7.27, *p* = 0.01; effect of time: *F*(3.949,118.5) = 16.4, *p* < 0.0001; Fig. 3e: two-way ANOVA: effect of housing condition: *F*(1,30) = 3.48, *p* = 0.07; effect of sex: *F*(1,30) = 7.27, *p* = 0.01; interaction: *F*(1,30) = 0.08, *p* = 0.78). Comparing the change in locomotor activity on the first day of habituation to the fifth day, male mice exhibited a decrease, regardless of housing condition (Fig. 3f: two-way ANOVA: effect of housing condition: *F*(1,16) = 2.09, *p* = 0.17, effect of test day: *F*(1,16) = 33.87, *p* < 0.0001). This change was also observed in our female mice (Fig. 3 g: two-way ANOVA: effect of housing condition: *F*(1,14) = 1.95, *p* = 0.18, effect of test day: *F*(1,14) = 6.55, *p* = 0.02). Just as in the light phase condition, if we run a three way ANOVA on this data, it reveals a significant test day by sex interaction with only the male mice exhibiting an effect of test day [test day x sex interaction: *F*(1,30) = 7.52, *p* = 0.01; Sidak’s post-hoc effect of day in group housed males: *p* = 0.0004; effect of day in socially isolated males: *p* = 0.009; effect of day in group housed females: *p* = 0.97; effect of day in socially isolated females: *p* = 0.35).

### Oxycodone-induced locomotor activity in the dark phase (Fig. 4a)

**Fig. 4:**
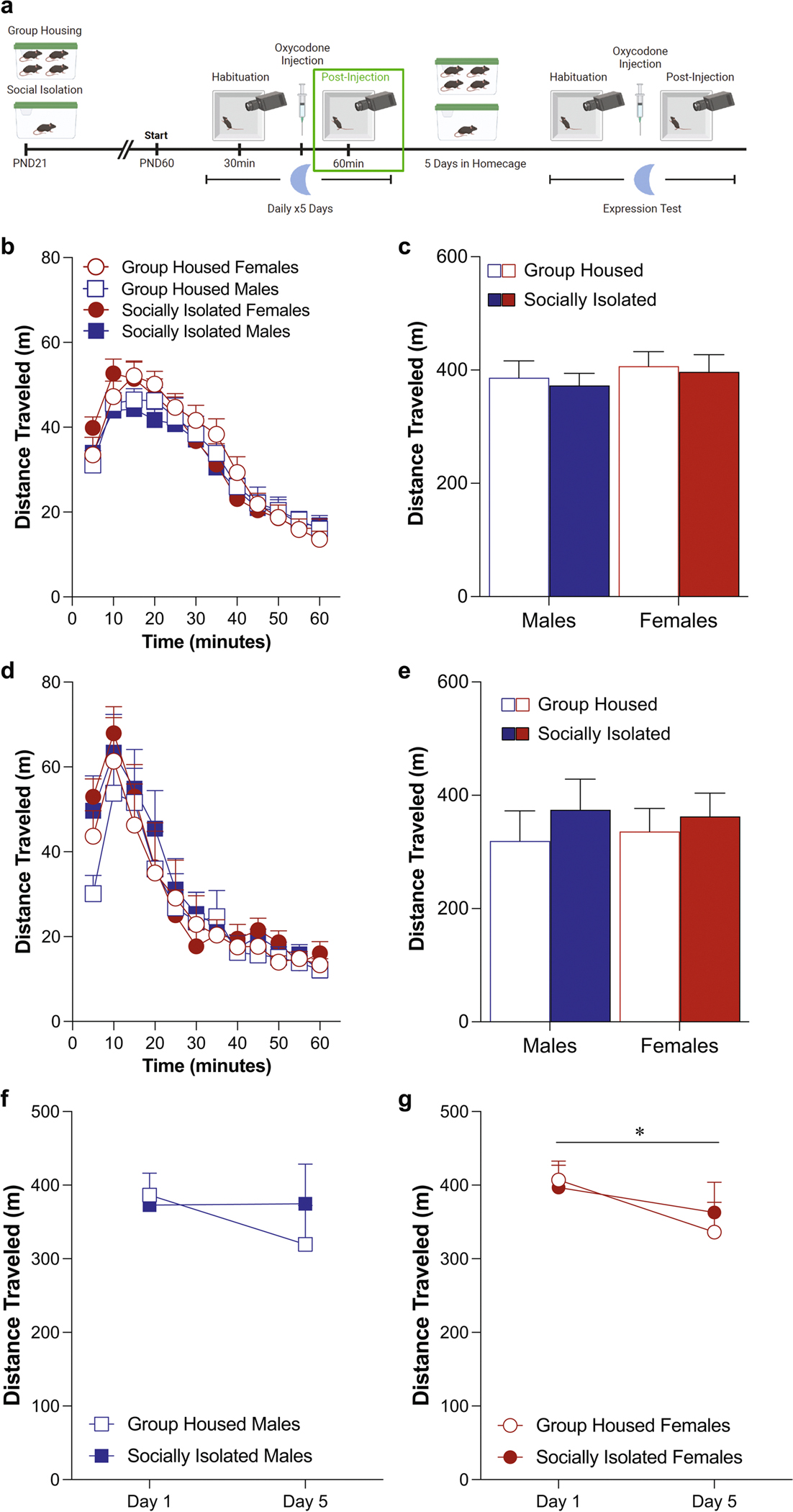
Female mice exhibit locomotor tolerance to oxycodone administered in the dark phase, while male mice do not. Schematic of experimental timeline with post-injection phase outlined (**A**). Adolescent social isolation does not alter the acute locomotor response to oxycodone (**B**, **C**). Adolescent social isolation does not alter the locomotor response after the fifth oxycodone administration (**D**, **E**). Male mice do not exhibit locomotor sensitization after five days of oxycodone (**F**). Female mice develop locomotor tolerance after five days of oxycodone (**G**, *p = 0.04). Locomotor activity is represented through distance traveled within five-minute bins (**B**, **D**) and total distance traveled (**C**, **E**). Data are presented as averages with SEM. (GH females *n* = 7, GH males *n* = 9, ASI females *n* = 9, ASI males *n* = 9)

When examining oxycodone-induced locomotor activity administered during the dark phase, we do not detect any effects of either housing condition or sex on Day 1 (Fig. 4b: three-way ANOVA: effect of housing condition: *F*(1,30) = 0.19, *p* = 0.67; effect of sex: *F*(1,30) = 0.66, *p* = 0.42; effect of time: *F*(3.238,97.14) = 105.7, *p* < 0.0001; Fig. 4c: two-way ANOVA: effect of housing condition: *F*(1,30) = 0.19, *p* = 0.67; effect of sex: *F*(1,30) = 0.66, *p* = 0.42; interaction: *F*(1,30) = 0.0004, *p* = 0.95). Similarly, there were no effects of housing condition or sex on oxycodone-induced locomotion after five days of oxycodone administration (Fig. 4d: three-way ANOVA: effect of housing condition: *F*(1,30) = 0.70, *p* = 0.41; effect of sex: *F*(1,30) = 0.002, *p* = 0.96; effect of time: *F*(3.446,103.4) = 52.8, *p* < 0.0001; Fig. 4e: two-way ANOVA: effect of housing condition: *F*(1,30) = 0.19, *p* = 0.67; effect of sex: *F*(1,30) = 0.66, *p* = 0.42; interaction: *F*(1,30) = 0.0004, *p* = 0.95). In contrast to what was seen when oxycodone was administered in the light phase, male mice did not exhibit sensitization after five days of oxycodone (Fig. 4f: two-way ANOVA: effect of test day: *F*(1,16) = 1.064, *p* = 0.32, effect of housing condition: *F*(1,16) = 0.17, *p* = 0.69). In contrast to the lack of change over time seen in female mice when oxycodone administered during the light phase, female mice receiving oxycodone during the dark phase developed tolerance after five daily injections (Fig. 4 g: two-way ANOVA: effect of test day: *F*(1,14) = 4.88, *p* = 0.04, effect of housing condition: *F*(1,14) = 0.03, *p* = 0.86).

### Habituation phase locomotor activity in light phase following forced abstinence (Fig. 5a)

**Fig. 5:**
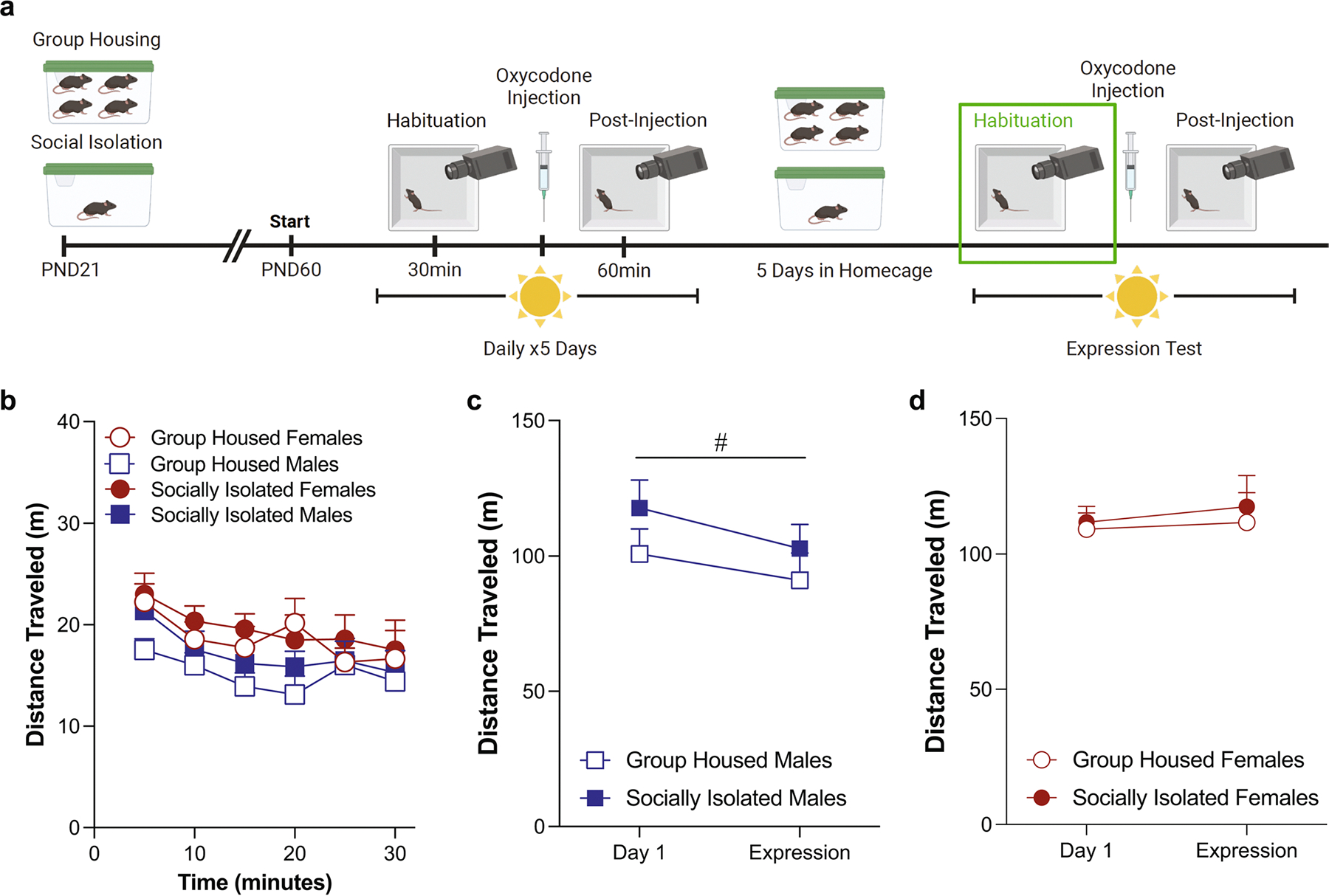
Neither male nor female mice exhibit alterations in locomotor behavior during the habituation phase following five days of forced abstinence. Schematic of experimental timeline with habituation phase outlined (**A**). Adolescent social isolation does not alter locomotor activity in the habituation phase on challenge day (**B**). Both male (**C**) and female (**D**) mice maintain baseline locomotor activity during expression day habituation phase. Male mice do appear to trend towards a decrease in locomotion on the fifth test day (**C**, #*p* = 0.07). Locomotor activity is represented through distance traveled within five-minute bins (**B**). Data are presented as averages with SEM. (GH females *n* = 5, GH males *n* = 5, ASI females *n* = 9, ASI males *n* = 9)

In a subset of animals previously tested during the light phase, we conducted additional tests of locomotor activity after a five-day forced abstinence to further examine the expression of oxycodone sensitization. All groups exhibited similar locomotor activity during the habituation phase including within session tolerance (Fig. 5b: three-way ANOVA: effect of housing condition: *F*(1,16) = 0.71, *p* = 0.41, effect of sex: *F*(1,16) = 2.89, *p* = 0.11; effect of time: *F*(5,80) = 11.10, *p* < 0.0001). When comparing locomotor activity during habituation on expression day to the initial locomotor response prior to any oxycodone administration, no differences were seen in male or female mice exposed to either housing condition (Fig. 5c: two-way ANOVA: effect of housing condition: *F*(1,8) = 1.38, *p* = 0.27, effect of test day: *F*(1,8) = 4.034, *p* = 0.07; Fig. 5d: two-way RM ANOVA: effect of housing condition: *F*(1,8) = 0.18, *p* = 0.68, effect of test day: *F*(1,8) = 0.25, *p* = 0.63).

### Oxycodone-induced locomotor activity in light phase following forced abstinence (Fig. 6a)

**Fig. 6:**
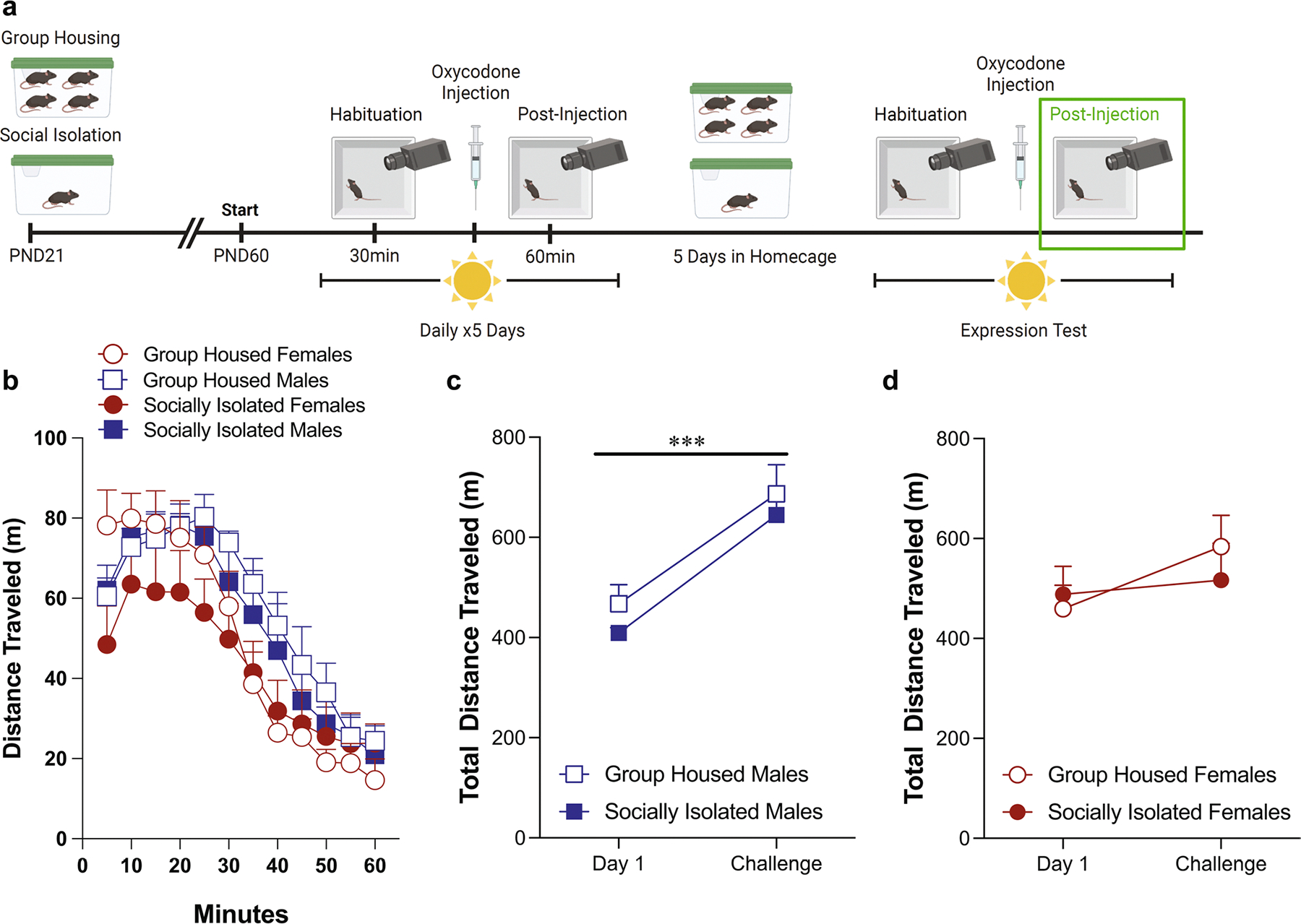
Male mice exhibit greater locomotor sensitization to light phase oxycodone following five days of forced abstinence. Schematic of experimental timeline with post-injection phase outlined (**A**). Adolescent social isolation does not alter the locomotor response to the final oxycodone challenge (**B**). Male mice exhibit sensitization after the final oxycodone challenge (**C**, ***p = 0.0005). Female mice do not exhibit sensitization after the final oxycodone challenge (**D**, *p* = 0.14). Locomotor activity is represented through distance traveled within five-minute bins (**B**). Data are presented as averages with SEM. (GH females *n* = 5, GH males *n* = 4, ASI females *n* = 9, ASI males *n* = 9)

When examining oxycodone-induced locomotor activity on the challenge day, we did not detect any effects of either housing condition or sex (Fig. 6b: three-way ANOVA: effect of housing condition: *F*(1,15) = 0.77, *p* = 0.40, effect of sex: *F*(1,15) = 3.42, *p* = 0.08; effect of time: *F*(2.767,41.50) = 67.28, *p* < 0.0001). When comparing oxycodone-induced locomotion on expression day to the response to the initial injection, both groups of male mice exhibited sensitization (Fig. 6c: two-way ANOVA: effect of housing condition: F(1,8) = 1.06, *p* = 0.33, effect of test day: *F*(1,8) = 30.68, *p* = 0.0005). In contrast, neither group of female mice exhibited sensitization (Fig. 6c: two-way ANOVA: effect of housing condition: *F*(1,7) = 0.06, *p* = 0.81, effect of test day: *F*(1,7) = 4.65, *p* = 0.07, interaction: *F*(1,7) = 1.83, *p* = 0.22).

### Habituation locomotor activity in the dark phase following forced abstinence (Fig. 7a)

**Fig. 7:**
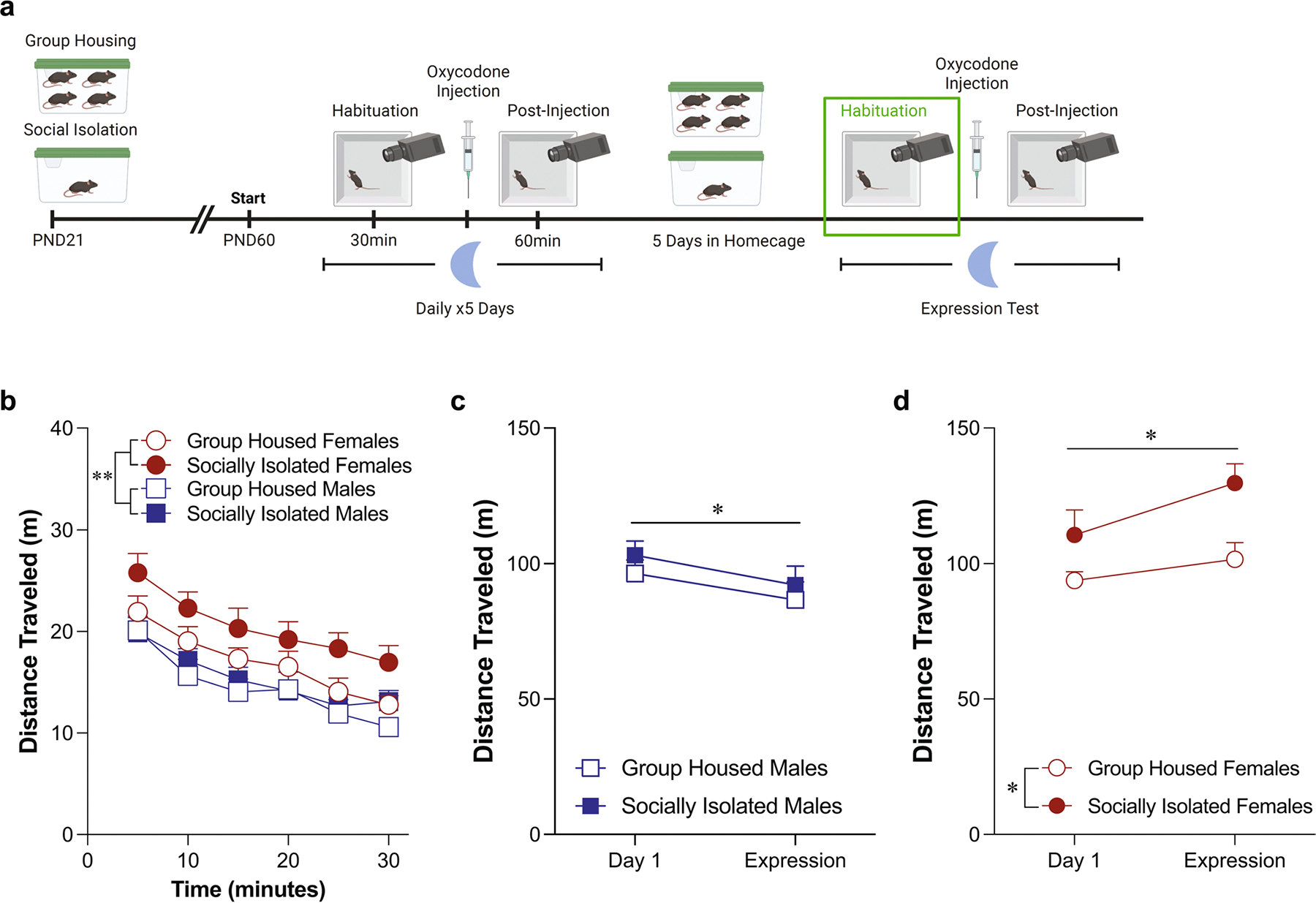
Sex differences in locomotor behavior during the habituation in the dark phase appear following five days of forced abstinence. Schematic of experimental timeline with habituation phase outlined (**A**). Female mice show greater locomotor activity compared to male mice during habituation phase after five days without testing (**B**, **p = 0.005). Male mice exhibit habituation after five days without testing (**C**, *p = 0.03). Female locomotor activity is sensitized during expression day habituation phase (**D**, *p = 0.02) while socially isolated females exhibit greater overall locomotion (**D**, *p = 0.02). Locomotor activity is represented through distance traveled within five-minute bins (**B**). Data are presented as averages with SEM. (GH females *n* = 7, GH males *n* = 9, ASI females *n* = 9, ASI males *n* = 9)

After a five-day forced abstinence period, all animals in the dark phase received additional tests of locomotor activity both prior to and after a final oxycodone challenge. During the habituation phase, female mice showed greater locomotor activity compared to males (Fig. 7b: three-way ANOVA: effect of sex: *F*(1,30) = 9.11, *p* = 0.005, effect of time: *F*(5,150) = 53.43, *p* < 0.0001, effect of housing condition: *F*(1,30) = 3.13, *p* = 0.09). When comparing the locomotor activity during habituation on expression day to the locomotor activity on day 1, male mice in both groups exhibit a habituated locomotor response (Fig. 7b: two-way ANOVA: effect of housing condition: *F*(1,16) = 0.70, *p* = 0.42, effect of test day: *F*(1,16) = 5.72, *p* = 0.03). In contrast, female mice exhibited a sensitized locomotor response on the final expression challenge day, indicative of a conditioned locomotor response with increased locomotion in socially isolated females (Fig. 7d: two-way ANOVA: effect of test day: *F*(1,13) = 6.46, *p* = 0.02). This effect was more pronounced in the socially isolated females [effect of housing condition: *F*(1,13) = 7.23, *p* = 0.02); interaction: *F*(1,13) = 1.14, *p* = 31).

### Oxycodone-induced locomotor activity in the dark phase following forced abstinence (Fig. 8a)

**Fig. 8:**
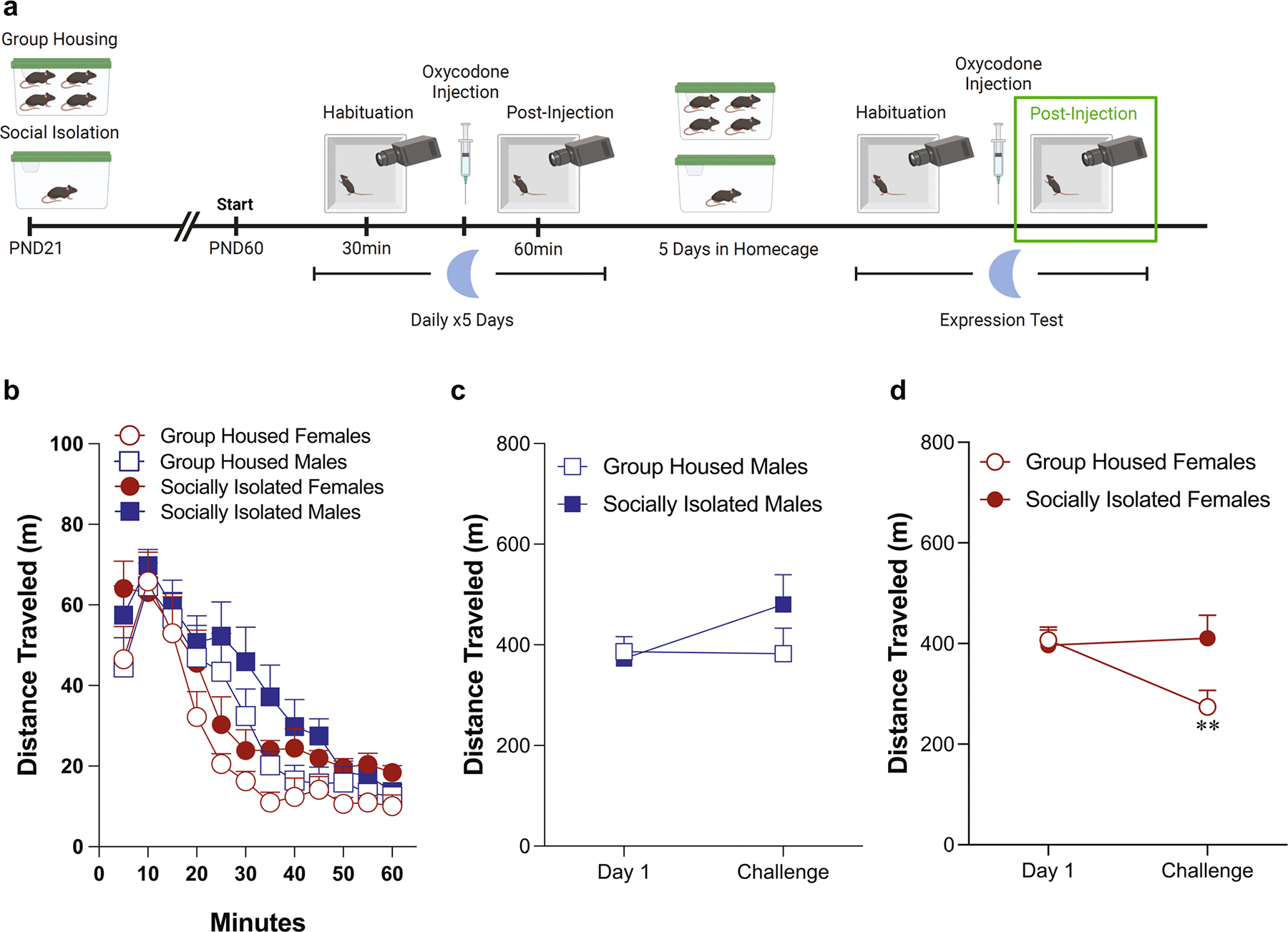
Adolescent social isolation alters oxycodone tolerance in female mice during the dark phase. Schematic of experimental timeline with post-injection phase outlined (**A**). Adolescent social isolation does not alter the locomotor response to the final oxycodone challenge (**B**). Male mice do not exhibit sensitization to the final oxycodone challenge (**C**). A Housing Condition X Sex interaction reveals that group housed female locomotor response to final oxycodone challenge in the dark phase demonstrates tolerance (**C**, **p = 0.009 (GH Females)). Locomotor activity is represented through distance traveled within five-minute bins (**B**). Data are presented as averages with SEM. (GH females *n* = 7, GH males *n* = 9, ASI females *n* = 9, ASI males *n* = 9)

Examining the time course of locomotion in the dark phase following the final oxycodone challenge reveals a significant time by sex interaction (Fig. 8b: three-way ANOVA: Time x Sex interaction: *F*(3.69,107) = 3.63, *p* = 0.0099, effect of time: *F*(3.69,107) = 71.48, *p* < 0.0001, effect of housing condition: F(1,29) = 4.05, *p* = 0.054, effect of sex: F(1,29) = 2.15, *p* = 0.15). When comparing locomotor activity on the challenge day to the first day of oxycodone administration, we did not see sensitization in male mice in either housing condition (Fig. 8c: males: two-way ANOVA: effect of housing condition: *F*(1,16) = 0.80, *p* = 0.38, effect of test day: *F*(1,16) = 1.75, *p* = 0.20). While we did not see sensitization in either group of female mice, group housed females show significant tolerance, exhibiting less oxycodone-induced locomotion on the challenge day (Fig. 8d: females: two-way RM ANOVA: effect of housing condition: *F*(1,14) = 2.06, *p* = 0.17, effect of test day: *F*(1,14) = 5.06, *p* = 0.04, interaction: *F*(1,14) = 7.61, *p* = 0.02; Sidak’s post-hoc group housed females Day 1 vs. Exp: *p* = 0.01).

## Discussion

Throughout our study we demonstrate differences in locomotor responses both prior to, and after, exposure to oxycodone. Throughout the habituation phase, we found sex differences that appear after the initial exposure to oxycodone and after a period of withdrawal in the dark phase. This points to long-lasting phase-dependent effects of oxycodone even in the absence of the drug. We found that the development of sensitization is both a sex-specific and light phase dependent process, as only our male mice were able to sensitize to oxycodone in the light phase. This provides insight into sex differences in contextual contributions to opioid responses that could potentially be mediated by circadian functions. We also observe the development of tolerance to oxycodone in female mice that occurred exclusively in the dark phase and only in group housed females following withdrawal. This further points to sex-specific contextual effects of oxycodone. Overall, we demonstrate the effect of adolescent social isolation on oxycodone behaviors primarily during the dark phase which provides important context for the further study of interactions between stress, contextual, and circadian factors. In addition to sex, we demonstrate the importance of considering time as an important methodological and physiological factor in the study of opioid behaviors as well as the consideration of biological sex in studying opioid responses.

### Oxycodone sensitization is light phase dependent in male mice

Male mice exhibit a sensitized locomotor response to oxycodone after five daily injections administered in the light phase. Following five days off drug, locomotor sensitization in response to our final oxycodone challenge administered during the light phase is potentiated in males. In contrast, when tested in the dark phase, male mice do not exhibit sensitization following either five daily injections or after the final challenge injection. The majority of drug-induced sensitization studies examine this behavior solely in the light phase presumably because sensitization develops more readily during the light phase (Abarca et al. 2002; Sell et al. 2002; Liu et al. 2005; Ericson et al. 2010; Niikura et al. 2013; Souza et al. 2014; Guegan et al. 2016; Perreau-Lenz et al. 2017; Libarino-Santos et al. 2020; Honeycutt et al. 2020; Hámor et al. 2023; Du et al. 2024). The light phase dependent sensitization we observed aligns with our previous findings and stimulant-induced behavioral sensitization studies documented in the literature (Gaytan et al. 1999, 2000; Hámor et al. 2023; Knouse et al. 2024). Taken together, this suggests that oxycodone sensitization is dependent upon the conditions or broader context such as the light phase in which the drug is administered.

The role of context in the development and expression of drug-induced sensitization has been documented previously. The testing environment, light phase, and behavioral cues from conspecifics can impact sensitization processes across drug classes. (Drew and Glick 1988; Badiani et al. 1995; Anagnostaras and Robinson 1996; Szumlinski et al. 1997; Arvanitogiannis et al. 2000; Battisti et al. 2000; Crombag et al. 2001; Anagnostaras et al. 2002; Araujo et al. 2006; Bloise et al. 2007; Yuan et al. 2022; Marinho et al. 2023; Coelho et al. 2025). These external cues contribute to the associative processes of sensitization. Interestingly, our results showing diurnal variation in sensitization aligns with a pattern observed in light phase dependent acquisition and consolidation of associative learning and memory. In rodents, fear conditioning acquisition is slowed or does not develop when training occurs during the dark phase (Chaudhury and Colwell 2002; Eckel-Mahan et al. 2008). Our repeated oxycodone exposure could represent a form of associative training, that, when executed during the dark phase, does not result in the intended outcome of sensitization. Sensitization is also thought to rely on the contribution of non-associative processes which can follow a similar diurnal acquisition pattern (Kuczenski et al. 1982; Anagnostaras and Robinson 1996; Anagnostaras et al. 2002) In non-associative learning, sensitization of the siphon-withdrawal reflex in *Aplysia* develops only when stimulus training occurred in the light phase (Fernandez et al. 2003). Identifying the neural mechanisms underlying both the associative and non-associative processes that contribute to the diurnal variation in sensitization and other drug behaviors could provide targets of further preclinical and clinical work. Given the diurnal nature of our results, the processes that contribute to sensitization could be related to the circadian molecular clock.

Our findings fit into the larger body of literature exploring circadian contributions to the development of sensitization processes. Shifting circadian rhythms with melatonin attenuates locomotor sensitization to cocaine or fentanyl, pointing to phase-specific molecular contributions to this process (Barbosa-Méndez et al. 2021; Du et al. 2024). Individual components of the light entrainment system might also contribute to the development of sensitization, as morphine sensitization does not occur when mu-opioid receptors are selectively knocked out of intrinsically-photosensitive retinal ganglion cells (Bergum et al. 2022). The expression of circadian clock genes *Per1*, *Per2*, and *Bmal1* have demonstrated a role in promoting or regulating cocaine and/or morphine sensitization (Abarca et al. 2002; Uz et al. 2003; Akhisaroglu et al. 2004; Perreau-Lenz et al. 2017; Castro-Zavala et al. 2022). Sensitization does not occur or is diminished in *Per1* deficient animals or when striatal PER1 levels are lowest (Akhisaroglu et al. 2004; Perreau-Lenz et al. 2017). The lowest levels of *Per1* expression have previously been reported to occur during the dark phase, which aligns with the testing timepoint at which our male mice did not sensitize to oxycodone (Akhisaroglu et al. 2004). Our findings support the evidence of circadian contributions to sensitization that could potentially translate to other drug behaviors. Pinpointing these mechanisms could further explore the already established link between opioid use disorders and circadian disruptions as a therapeutic target.

### Female mice do not exhibit sensitization to oxycodone

The current study found that female mice did not exhibit sensitization to 5 mg/kg of oxycodone in either the light or dark phase. The ethogram of opioid-related behaviors in mice is largely dependent on observed behaviors in males. Female animals have traditionally been excluded from preclinical studies, thus most studies examining sensitization solely include male animals (Gaytan et al. 1999, 2000; Abarca et al. 2002; Liu et al. 2005; Niikura et al. 2013; Yu et al. 2015; Guegan et al. 2016; Perreau-Lenz et al. 2017; Bergum et al. 2022; Du et al. 2024). This gap in the literature makes it difficult to predict if male and female animals can and will sensitize to the same stimulus. Our results highlight the sex differences in the effects of long-term drug exposure and sensitization processes. For example, female mice develop sensitization to ethanol, nicotine, amphetamine, and cocaine in specific contexts (Robinson 1984; Weiss et al. 2001; Sell et al. 2002; Ericson et al. 2010; Souza et al. 2014; Hamilton et al. 2014; Libarino-Santos et al. 2020; Honeycutt et al. 2020). While we did not detect sensitization in the current study this may suggest that the dose response curve for this behavioral phenotype is different in females. Previous studies in rats suggest that females may respond more for higher doses of oxycodone (Zanni et al. 2020; Sharp et al. 2021), so perhaps we would have seen sensitization to a higher dose of oxycodone. However, our results may also suggest that the behavioral phenotypes seen following oxycodone intake are sex-dependent and overall the assumption that behavioral phenotypes observed in males will be consistent in females is not valid.

The lack of sensitization in our female mice also highlights the role of context and novelty in the development of sensitization. The degree of sensitization and conditioning is largely dependent on the environment in which the drug is given, either novel or familiar (Badiani et al. 1995; Szumlinski et al. 1997; Battisti et al. 2000; Crombag et al. 2001; Coelho et al. 2011, 2025; Ferreira et al. 2020) Previously, male rats have been shown to only sensitize to morphine in a novel environment while exhibiting a tolerance-like effect in a familiar environment (Coelho et al. 2025). The trend of our female mice to not sensitize in the light phase and develop tolerance in the dark phase could demonstrate that the female mice are not interpreting the testing environment or testing within the dark phase as novel. Additionally, our female mice do not show a typical habituation effect in locomotor activity during the habituation phase in either the light or dark phase which also points to a sex-specific response to novelty. In this case, our male mice possess a vulnerability to novelty and novel environments that could produce greater susceptibility to sensitized opioid behaviors.

The difference in oxycodone-induced locomotion between our male and female mice also follows previous studies that found sex differences in sensitized stereotypy. Sensitized behaviors in female rodents are frequently characterized as stereotypies and differ from gross horizontal locomotor activity used in the current study (Kilbey and Ellinwood 1977). It is possible that sensitization in our female mice manifests through different behaviors beyond locomotor activity represented as distance traveled. Our findings and the literature point to sex differences in the behavioral manifestation of long-term oxycodone exposure and the need to expand the ethogram to further characterize behaviors that could differ between the sexes. Identifying these differences and any sex-specific behaviors could also highlight the underlying mechanisms of sensitization and provide targets for future studies.

### Tolerance to the locomotor stimulating effects of oxycodone is light phase dependent and is influenced by adolescent social isolation

When tested during the dark phase, female mice developed tolerance to the locomotor stimulating effects of oxycodone between the first and fifth day. After the final dark phase oxycodone challenge, group housed female mice continued to exhibit tolerance. This tolerance to the locomotor activating effects of oxycodone in group housed female mice is consistent with previous findings in our lab that demonstrated that wildtype female mice exhibit a similar decrease in response to repeated oxycodone exposure in the dark phase (Knouse et al. 2024). There is an established basis for the context-dependent development of tolerance to ethanol in male rats during the light phase (Bueno and Fachini 2007). Given that the sex-specific effects found in our work are only observed in the dark phase, we have potentially uncovered a context-specific vulnerability for our female control mice to develop long-lasting tolerance.

Socially isolated female mice developed tolerance to the locomotor stimulating effects of oxycodone between the first and fifth day when exposed during the dark phase. However, following the final oxycodone challenge, our socially isolated females exhibited baseline locomotor responses like that of their acute oxycodone exposure. During the dark phase, the effects of social isolation after withdrawal may sensitize the previous response or prohibit lasting plasticity that would promote significant sensitization or tolerance. During the light phase, adolescent social isolation might also prohibit long-term behavioral plasticity in females as no change in day was seen throughout the three reported timepoints.

### Sex differences in locomotor behavior during the habituation phase

While typically animals habituate to a novel environment and decrease their locomotor activity over time, this occurred less in our female mice across the testing conditions. On the fifth day of habituation, female mice exhibit increased locomotor activity compared to males in the dark phase. Further, the change in locomotor activity during habituation over the course of the five days was greater in males in both the light phase and the dark phase and on the expression test day females exhibited even higher rates of locomotion. As female mice in both groups exhibit similar levels of within session habituation on the first day of testing, we conclude that oxycodone exerts a unique lasting effect on female mice. This increased activity could be attributed to the conditioning properties of oxycodone that has resulted in anticipatory locomotor activity in response to returning to the drug-associated environment. Conditioned behaviors could contribute to susceptibility of continued administration and drug seeking (Foltin and Haney 2000; Alleweireldt et al. 2001). Our results could demonstrate a sex-specific sensitivity to the conditioning effects of oxycodone that may promote a drug seeking vulnerability in female mice. The increase we observed in female mice could also be a result of sustained locomotion in a novel environment. The response to novelty is heavily influenced by mu-opioid manipulation and differs by sex between scenarios (Koos Slob et al. 1986; Van Haaren and Meyer 1991; Veenema et al. 2012; Smith et al. 2015; Liang et al. 2017; Kuhn et al. 2022). Differing responses to novelty could promote drug taking and seeking. Regardless of the mechanism contributing to this difference, female mice are behaving differently than males in response to returning to the drug-associated environment, which may indicate sex differences in behavioral plasticity. The potential vulnerability we observed could be increased following withdrawal in a light-phase specific manner. In the habituation phase on challenge day, female mice have increased locomotor activity compared to males only during the dark phase. This could indicate a potential sensitivity to cue re-exposure that may facilitate greater drug seeking behaviors in female mice. While no sex differences have currently been found in relapse behaviors in rats, the sex difference we observe in locomotion following withdrawal warrants further study (Grimm et al. 2001; Venniro et al. 2019; Reiner et al. 2020).

## Conclusions

Our results indicate that adolescent social isolation stress alters oxycodone behaviors in a light phase dependent manner with its effects mostly manifesting during the dark phase testing. This poses an important consideration for the methodology behind stress studies and the contexts in which the long-term effects of stress manifest. Our results concerning habituation and sensitization to a drug-associated context also demonstrate baseline bidirectional sex differences in behavioral drug-induced plasticity. Further, our results indicate baseline sex and light phase differences in the sensitization process which provides important context for previous models of drug-induced sensitization.

## Supplementary Material

Supplemental Figure 1

Supplemental Figure 2

**Supplementary Information** The online version contains supplementary material available at https://doi.org/10.1007/s00213-025-06914-8.

## Data Availability

The datasets used and/or analyzed during the current study are available from the corresponding author on request.
