# Supplementary figures and images for: Examining the impact of adolescent social isolation on oxycodone sensitization
Source: Psychopharmacology (Berl). Author manuscript; Available in PMC 2026 Feb 12. (PMC12892223; doi:10.1007/s00213-025-06914-8)

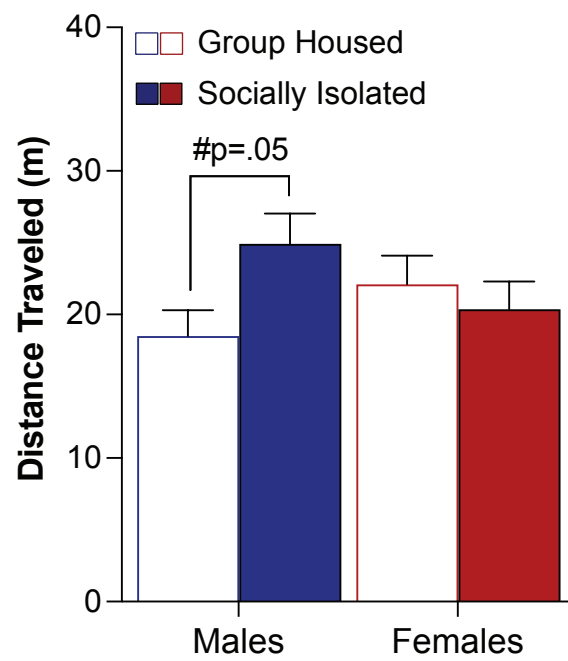

Supplement: Supplemental Figure 1 [file NIHMS2135060-supplement-Supplemental_Figure_1.pdf]

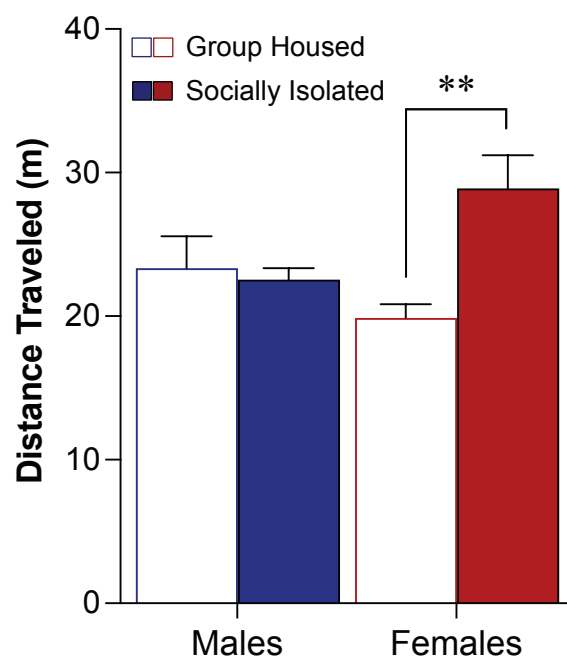

Supplement: Supplemental Figure 2 [file NIHMS2135060-supplement-Supplemental_Figure_2.pdf]
